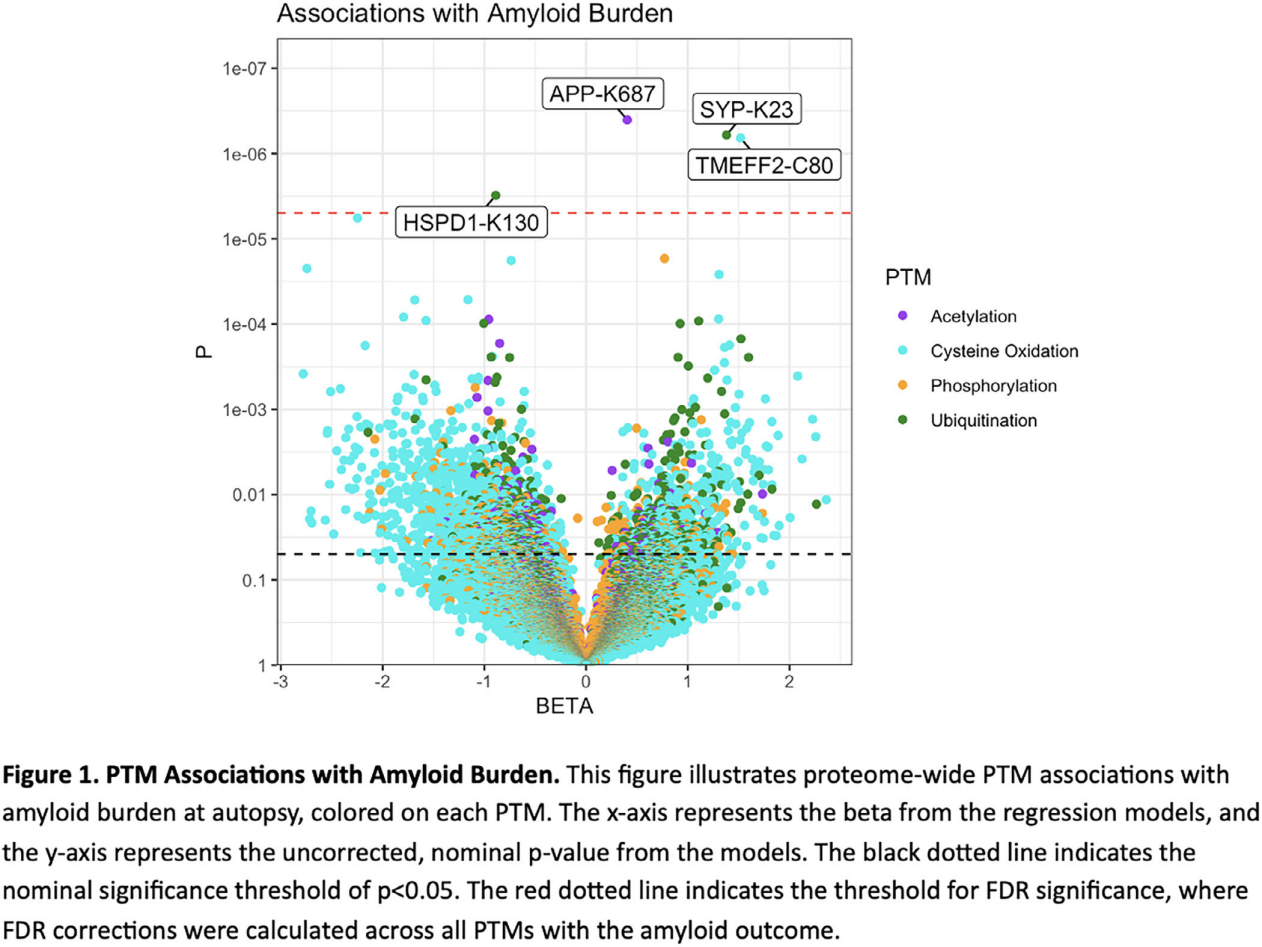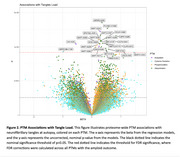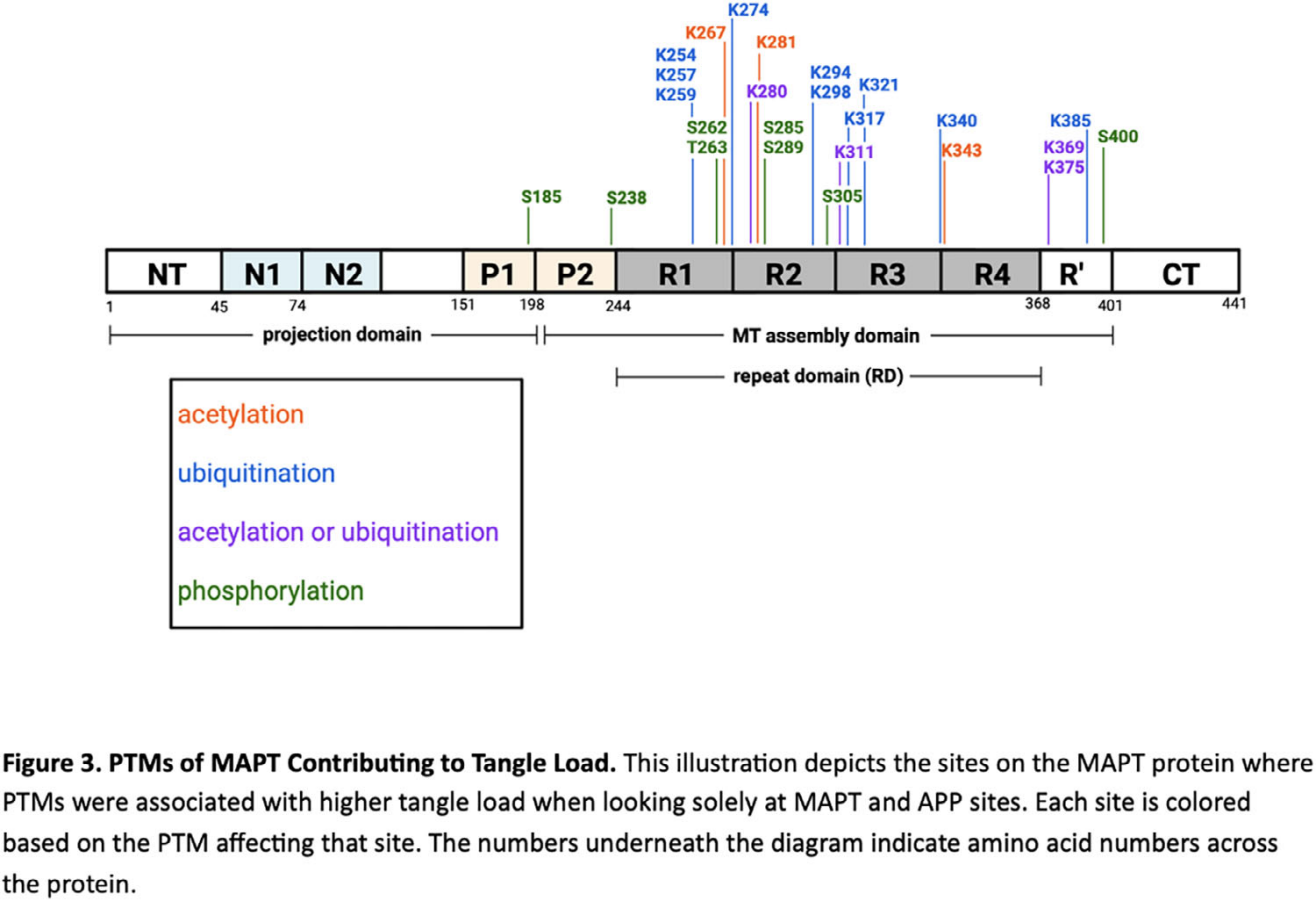# post translational modifications in the brain are critical contributors to Alzheimer's disease neuropathology and cognitive decline

**DOI:** 10.1002/alz70855_105864

**Published:** 2025-12-24

**Authors:** Julia B. Libby, Philip L. De Jager, Vilas Menon, Shahram Oveisgharan, Julie A Schneider, Lisa L. Barnes, David A. A. Bennett, Vladislav A Petyuk, Timothy J. Hohman

**Affiliations:** ^1^ Vanderbilt Memory & Alzheimer's Center, Vanderbilt University Medical Center, Nashville, TN, USA; ^2^ Cell Circuits Program, Broad Institute, Cambridge, MA, USA; ^3^ Center for Translational & Computational Neuroimmunology, Columbia University Irving Medical Center, New York, NY, USA; ^4^ Rush Alzheimer's Disease Center, Rush University Medical Center, Chicago, IL, USA; ^5^ Biological Sciences Division, Pacific Northwest National Laboratory, Richland, WA, USA; ^6^ Department of Neurology, Vanderbilt Memory & Alzheimer's Center, Vanderbilt University Medical Center, Nashville, TN, USA; ^7^ Vanderbilt Memory and Alzheimer's Center, Vanderbilt University School of Medicine, Nashville, TN, USA

## Abstract

**Background:**

Proteins undergo various modifications after translation, some of which are known to contribute to AD. For example, post‐translational modifications (PTMs) in APP and MAPT contribute to plaques and tangles in Alzheimer's disease (AD). Yet, little is known about the contributions of such PTMs across the proteome in the AD brain. Therefore, this proteome‐wide study identifies PTMs in the AD brain that contribute to AD neuropathology and cognitive decline.

**Method:**

PTMs were quantified with mass spectrometry leveraging prefrontal cortex tissue from 34 cognitively normal, 33 MCI, and 34 AD dementia participants from the Religious Orders Study and Rush Memory and Aging Project (ROS/MAP). 51,654 PTMs across 9,829 proteins were quantified, including acetylation (acet), ubiquitination (ubi), phosphorylation (phos), and cysteine oxidation (cyst). Outcomes included neuropathologically‐confirmed AD dementia diagnosis, immunohistochemistry measurements of tau tangle density and β‐amyloid load, and cross‐sectional and longitudinal measures of global cognition. Covariates included sex, age at death, clinical diagnosis, *APOE*‐ε4, and post‐mortem interval. Correction for multiple comparisons leveraged the false discovery rate (FDR) procedure.

**Result:**

Multiple known PTM sites of MAPT contributed to tau tangle density including acet‐K343 (β=0.29, P.FDR=0.01), ubi‐K298 (β=0.32, P.FDR=0.02), phos‐S285 (β=0.24, P.FDR=0.02), phos‐S289 (β=0.27, P.FDR=0.02), phos‐S238 (β=0.19, P.FDR=0.02), acet‐K311 (β=0.25, P.FDR=0.03), and phos‐S262 (β=0.13, P.FDR=0.03). We also observed many novel PTM associations. Interestingly, acetylation of APP‐K687, a known site of autosomal dominant AD mutations, was associated with higher amyloid burden (β=0.40, P.FDR=0.01). Ubiquitination of SYP‐K23 (β=1.38, P.FDR=0.01) and cysteine oxidation of TMEFF2‐C80 (β=1.52, P.FDR=0.01) also related to a higher amyloid burden, while ubiquitination of HSPD1‐K130 related to lower burden (β=‐0.89, P.FDR=0.04). In tau analyses, ubiquitination of ARFIP‐K290 (β=0.35, P.FDR=0.009), PLXND1‐K1826 (β=0.53, P.FDR=0.01), and QPRT‐K288 (β=0.83, P.FDR=0.02) related to higher tangle burden. Finally, ubiquitination of both LDHB‐K91 (β=‐0.74, P.FDR=0.02) and TAP2‐K245 (β=‐0.75, P.FDR=0.02), and cysteine oxidation of RAI1‐C1400 (β=2.00, P.FDR=0.04), related to cross‐sectional global cognitive performance.

**Conclusion:**

This proteome‐wide examination of PTMs in the AD brain highlight known and novel PTMs in MAPT and APP, while also identifying novel protein PTMs related to amyloid, tau, and cognitive impairment. These results indicate vast changes to proteins during AD and highlight numerous novel targets for intervention.